# The Means by Which the Temperature of the Body Is Maintained in Health and Disease

**Published:** 1897-07-03

**Authors:** W. Hale White

**Affiliations:** Physician to, and Lecturer on Pharmacology at, Guy's Hospital


### Medical Progress and Hospital Clinics.

[*The Editor will be glad to receive offers of co-operation and contributions from members of the profession. All letters should be addressed to* The Editor, at the Office, 28 & 29, Southampton Street, Strand, London, W.C.]

### THE MEANS BY WHICH THE TEMPERATURE OF THE BODY IS MAINTAINED IN HEALTH AND DISEASE.

The Croonian Lectures, delivered before the Royal College of Physicians of London.

By W. Hale White, M.D., F.R.C.P. Lond., Physician to, and Lecturer on Pharmacology at, Guy's Hospital.

### Lecture III.

This lecture was principally devoted to a description of the various forms of calorimeters which have been used for estimating the amount of heat produced by animals. The problem might at first sight seem simple, but in reality it was a very complicated one. Not only did an animal give off heat by radiation and convection, but a large amount of the heat which it produced was rendered latent by evaporation from the skin and air passages. The increase of the watery vapour in the air had, therefore, to be observed, and the amount of latent heat calculated from it. The varying temperature of the outer air, convection and radiation from the instrument, and the difficulty of ensuring proper mixing of the material by which the heat was absorbed, be it air or water, so as to obtain a true average, were all mentioned as complications adding to the difficulty of the problem.

Calorimeters might be roughly divided into two classes. In one class it had to be determined, by previous experiment with known sources of heat, how many calories were required to produce a given rise, *i e.*, the instruments had to be previously standardised by the use of some such source of heat as the burning of a given quantity of hydrogen, or the cooling of a vessel containing a given quantity of hot water, and the results of experiments with animals were worked out according to the scale so laid down. In the other class, which Dr. Hale White greatly preferred, the apparatus was duplicated, the two sides being exact copies of one another, and while the animal was placed in one, a measurable source of heat, such as a hydrogen flame, was placed in the other. By this "differential" method many chances of error were removed. Dr. Hale White then described the apparatus which he had devised.

[Leaving out of consideration all details as to the arrangements for preventing loss of heat, ensuring proper ventilation, lighting the lamp, condensing the vapour, &c., the apparatus may thus be described: Two air-tight chambers are prepared, in one of which is placed a hydrogen flame capable of regulation, in the other the animal to be observed. These two chambers, which are made of thin metal, are double-walled, the interval between the inner and outer wall forming in each an air-tight cavity. These cavities communicate the one with the other by means of a piece of glass tubing bent down in the middle, where it is filled with oil, thus forming a manometer. These two double jackets and the tube connecting them practically form a large differential thermometer, and the slightest increase of the temperature of one over that of the other will expand the air within it to a greater degree and will drive the oil in the manometer towards the opposite side. So long, then, as the manometer remains steady, we may be sure that neither jacket is receiving more heat than the other. By regulating the flame, then, which is in the chamber enclosed by the one jacket so that the manometer remains level, and calculating the amount of heat produced by the amount of hydrogen used, we may tell with great exactitude the amount of heat which is being given up by the animal to the jacket of the other chamber. By then drawing out the air from this chamber through an apparatus, by which the watery vapour produced can be absorbed and weighed, it is possible to calculate with great nicety how much heat is to be added on account of evaporation from the lungs and skin. The total amount of heat lost by the animal in a given time can thus be obtained.]

Dr. Hale White next proceeded to show that, although by the apparatus he had described it was possible to measure the amount of heat given off by the animal, both by radiation, conduction, and evaporation, it had to be remembered that the standard of comparison was a flame by which heat was steadily produced and given off, whereas the animal had within itself the power to vary not only the speed of heat production, but the degree to which heat was given off by the skin. This, he thought, introduced a fallacy. A much more serious difficulty arose from the very different manner in which the heat was distributed in the chamber according as it came from the small intense point produced by a flame of burning hydrogen, and the large and comparatively cool, hairy surface of the animal. To sum up, Dr. White considered that (*a*) no animal calorimeter that held out any prospect of being workable could give exactly the amount of heat produced by an animal, for when it showed an alteration in the number of calories lost it did not show in what proportion this alteration was due to change in the amount of radiation and in what to change in the amount of production. But if we knew the internal temperature of the animal, its weight, and its specific heat, we could often say whether the production of heat was increased or diminished. In other words we could get a qualitative although not an accurate quantitative result. (*b*) It was necessary to be very careful in drawing conclusions in regard to man from experiments on animals in consequence of the fact that variations in temperature might depend on variations in loss of heat by the skin in much larger degree in man than in furry animals. (*c*) A differential instrument was better than one which had to be previously standardised. (*d*) The source of heat in a differential instrument should resemble in shape the animal which was to be observed. Finally, he again insisted on the fact that the reason of a rise of temperature varied in different fevers and in different animals. In some fevers the rise of temperature was chiefly due to variations in the production of heat, in others it was chiefly due to variations in the loss, and experiments could only hold good for the particular animal and the particular fever under consideration; moreover, it must be remembered that however perfect our thermometric observations might be, no calorimeter could register heat which was converted into some other form of energy in the body—for example into motion.

